# ChatGPT Interactive Medical Simulations for Early Clinical Education: Case Study

**DOI:** 10.2196/49877

**Published:** 2023-11-10

**Authors:** Riley Scherr, Faris F Halaseh, Aidin Spina, Saman Andalib, Ronald Rivera

**Affiliations:** 1 Irvine School of Medicine University of California Irvine, CA United States; 2 Department of Emergency Medicine Irvine School of Medicine University of California Irvine, CA United States

**Keywords:** ChatGPT, medical school simulations, preclinical curriculum, artificial intelligence, AI, AI in medical education, medical education, simulation, generative, curriculum, clinical education, simulations

## Abstract

**Background:**

The transition to clinical clerkships can be difficult for medical students, as it requires the synthesis and application of preclinical information into diagnostic and therapeutic decisions. ChatGPT—a generative language model with many medical applications due to its creativity, memory, and accuracy—can help students in this transition.

**Objective:**

This paper models ChatGPT 3.5’s ability to perform interactive clinical simulations and shows this tool’s benefit to medical education.

**Methods:**

Simulation starting prompts were refined using ChatGPT 3.5 in Google Chrome. Starting prompts were selected based on assessment format, stepwise progression of simulation events and questions, free-response question type, responsiveness to user inputs, postscenario feedback, and medical accuracy of the feedback. The chosen scenarios were advanced cardiac life support and medical intensive care (for sepsis and pneumonia).

**Results:**

Two starting prompts were chosen. Prompt 1 was developed through 3 test simulations and used successfully in 2 simulations. Prompt 2 was developed through 10 additional test simulations and used successfully in 1 simulation.

**Conclusions:**

ChatGPT is capable of creating simulations for early clinical education. These simulations let students practice novel parts of the clinical curriculum, such as forming independent diagnostic and therapeutic impressions over an entire patient encounter. Furthermore, the simulations can adapt to user inputs in a way that replicates real life more accurately than premade question bank clinical vignettes. Finally, ChatGPT can create potentially unlimited free simulations with specific feedback, which increases access for medical students with lower socioeconomic status and underresourced medical schools. However, no tool is perfect, and ChatGPT is no exception; there are concerns about simulation accuracy and replicability that need to be addressed to further optimize ChatGPT’s performance as an educational resource.

## Introduction

After decades of development, artificial intelligence (AI) is an increasingly common talking point in medicine. AI (ie, computer systems capable of advanced functions like writing, vision, data analysis, and speech recognition) has a host of potential applications across the clinical and research spectra, including drafting clinical documentation, reading imaging studies, and expediting literature reviews [1]. However, AI might change more than how medicine is practiced; it may change how medicine is taught. Specifically, AI chatbots, such as OpenAI’s ChatGPT [2], have the potential to improve medical education. Equipped with swift efficacy and memory, remarkable accuracy, and a personable interactive style, ChatGPT can execute complex and creative tasks [3]. ChatGPT has already entered the medical education arena, having passed curated, publicly available versions of the United States Medical Licensing Examination (USMLE) Step 1, Step 2 Clinical Knowledge, and Step 3 questions earlier this year [4]. Its applications in medical education are just beginning to be discovered with emerging research and increased use by medical students; possible uses range from facilitating research projects and creating study guides and flashcards to enhancing textbook explanations [5,6].

Despite a widespread movement to incorporate clinical experiences early and longitudinally in preclinical years, these two main phases of medical education are fundamentally different [7]. The preclinical curriculum teaches the scientific foundations of medicine, while the clinical curriculum synthesizes and enhances this foundational information so that it can be applied to patient care. As a result, students go from a world of controlled, direct lines of inquiry into a world of great variability. Medical education has tried to flatten this learning curve; for example, the USMLE Step 1 exam at the end of preclinical years often frames questions as clinical vignettes to encourage clinical thinking, and many medical schools have simulation centers for students. Still, the transition remains difficult. One study shows that 87% of medical students transitioning to clinical clerkships worry that they have significant knowledge gaps between basic science pathophysiology and diagnostic reasoning [8]. Although simulation centers could be used more to address these concerns, running a simulation requires coordinating schedules, expensive equipment, script writing, and other logistics [9]. Cost is especially relevant (and potentially prohibitive) in the context of global medical education; not all US medical schools—let alone medical schools in less wealthy nations—can afford simulation centers. Thus, increasing simulation centers alone is an impractical and potentially inequitable solution to a complex issue. Alternatively, other web-based simulation resources can help students with clinical exposure, such as the computer-based case simulations Step 3 Case Simulator. This simulation bank is relatively inexpensive with exceptional realism designed to prepare residents for Step 3 [10]. However, this resource has a very limited number of simulations and is far above the educational level of most medical students, making it unsuitable for the preclinical to clinical transition. An additional cost-effective simulation tool for practicing diagnostic and clinical reasoning would thus be welcomed, and ChatGPT has been identified as a possible solution.

This paper offers transcribed conversations with ChatGPT as an example of how its interactive clinical simulations can help bridge preclinical and clinical training. We present 3 different examples of interactive clinical simulations generated by ChatGPT, each modified slightly to highlight different capabilities.

## Methods

To start, we selected two simulation categories with which to run prompts in ChatGPT. We chose advanced cardiac life support (ACLS) and 2 medical intensive care unit (ICU) scenarios—pneumonia and sepsis; ACLS was chosen because of the ACLS algorithm’s simplicity, while the medical ICU category was chosen for its potential complexity. Both simulations were inspired by Irvine School of Medicine’s “Clinical Foundations I” simulation curriculum at the University of California [11]. 

**Figure 1:**
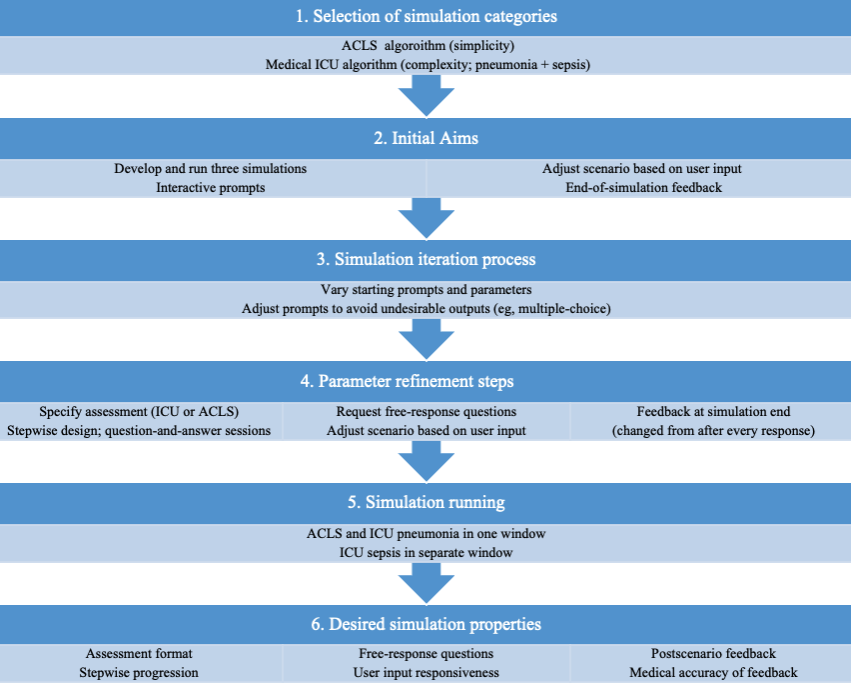
Study design flow. ACLS: advanced cardiac life support; ICU: intensive care unit.

A free account was made with OpenAI to run ChatGPT 3.5 on Google Chrome (version 133.0.5672.126). We aimed to prompt ChatGPT to create and run 3 simulations from beginning to completion. Our goal was an interactive simulation that periodically asked the user how they wanted to treat the patient, adjusting the scenario based on the user’s open-ended responses and summarizing performance feedback at the end of the simulation. Simulations were created and run by trialing varying starting prompts and scenario parameters until ChatGPT produced an undesirable output (eg, a multiple-choice question rather than a free response). Starting prompts were changed for the next attempted simulation based on the type of error or lack of a simulation parameter. A flowchart of our methods can be found in Figure 1.

As we refined our input, we learned that we had to present several parameters to ChatGPT to achieve our goal through this stepwise approach. The initial step was to tell ChatGPT the type of assessment we wanted, specifying the scenario as ICU or ACLS. Next, we requested that the scenario be run in a stepwise nature with question-and-answer sessions at each stage of the scenario. We then learned that we had to specify the question type as a free-response question so that multiple-choice questions were not used. We then learned that we needed to tailor ChatGPT’s response to the questions by asking it to adjust the scenario based on the user’s free-form responses to the question. This meant that the scenario had to improve or worsen the patient’s condition based on the user’s input. At this step, the software was giving feedback about the correctness of user responses after each question. We felt that this made the scenarios too simplistic and not representative of real-world situations, so we added a final command to address feedback timing. We specified that we wanted feedback about the answers only at the end of the simulation. Table 1 presents the breakdown of one ICU scenario. ACLS Simulation (Textbox 1) and ICU Pneumonia (Textbox 2) were run in the same conversation window. ICU Sepsis (Textbox 3) was run in a different conversation window for cleaner data keeping. All simulations were run by the first author (RS); medical accuracy was assessed by all authors, especially by RR. RR is a board-certified emergency medicine physician credentialed in ACLS and advanced trauma life support. He is also a curriculum designer for a large emergency medicine residency program and associated medical school with 5 years of experience designing curricula. He has the requisite experience and knowledge to review the answers for veracity and accuracy.

**Table 1:** Development of starting prompts. Columns identify the desired simulation parameter and associated input for a test simulation. Rows identify the given test simulation. Italicized phrases indicate changes between similar responses.

Test simulation	Assessment	Stepwise nature	Question type	Responsive to user inputs	Feedback timing
Test simulation 1	Please create a medical ICU^a^ scenario….	…and quiz me on the proper next step of management.	NA^b^	NA	NA
Test simulation 2	Please create a medical ICU scenario….	…and quiz me on the proper next step of management.	Please make the quiz free response….	Please make the quiz…interactive, meaning you ask me what the next step is, and then I write out what I would do, and then you ask me another question based on how my answer would affect the patient?	NA
Test simulation 3	Please create a medical ICU scenario….	…and quiz me on the proper next step of management.	Please make *it* free response….	Please make *it*…interactive, meaning you ask me what the next step *is one question at a time*, and then I write out what I would do, and then you ask me another question based on how my answer would affect the patient….	…and don’t tell me the right answers until the end of the scenario

^a^ICU: intensive care unit.

^b^NA: not asked.

**Advanced cardiac life support (ACLS) Simulation. Sample interactive clinical simulation of an ACLS case. Bolded text represents user responses. Italicized text represents ChatGPT responses. The initial bolded text represents the initial simulation prompt inputted by the user. Multimedia Appendix 1 contains the full-length scenario.**

**Please create an ACLS scenario and quiz me on what the proper next step of management is. Please make it free response and interactive, meaning you ask me what the next step is one question at a time, and then I write out what I would do, and then you ask me another question based on how my answer would affect the patient. Please update / change the patient’s condition based on my actions, and do not tell me the right answers until the end of the scenario.**

Sure! Here’s an ACLS (Advanced Cardiovascular Life Support) scenario for you:

Scenario:

You are a healthcare provider working in the emergency department, and a 58-year-old male patient is brought in by ambulance. The patient is unresponsive, not breathing, and has no pulse. The patient’s history includes a known history of coronary artery disease, and the ECG monitor shows a wide-complex tachycardia without a pulse. The team initiates ACLS protocols.

*See Multimedia Appendix 1 for full text.*

What would be your next step in managing this patient?

Your response here

**Identify the ECG finding as a shockable rhythm. defibrillate patient after confirming all personnel are clear.**

Great! You identify the wide-complex tachycardia as a shockable rhythm. You ensure that all personnel are clear, and then you deliver a defibrillation shock to the patient.

Question 3:

After the defibrillation, the rhythm on the ECG monitor shows asystole. What would be your next step in managing this patient?

[Your response here]

**Continue CPR and ventilation. Establish IV access and administer 1 mg epinephrine every 3-5 minutes. Stop every 2 minutes for pulse check**.

*See Multimedia Appendix 1 for full text.*

Based on your responses, you provided appropriate ACLS interventions for a patient in cardiac arrest. However, it is important to note that ACLS guidelines are constantly evolving, and the appropriate management of a specific patient may vary based on their individual medical history and other factors. It is also important to remember that effective communication and teamwork among all members of the resuscitation team is crucial for optimal patient outcomes.

**Intensive care unit (ICU) Pneumonia. Sample interactive clinical simulation of a medical ICU case. Bolded text represents user responses. Italicized text represents ChatGPT’s responses. The initial bolded text represents the initial simulation prompt inputted by the user. Multimedia Appendix 2 contains the full-length scenario.**

**Please create a medical ICU scenario and quiz me on what the proper next step of management is. Please make it free response and interactive, meaning you ask me what the next step is, and then I write out what I would do, and then you ask me another question based on how my answer would affect the patient. Please update / change the patient’s condition based on my action, and do not tell me the right answers until the end of the scenario.**

Certainly! Here’s a medical ICU scenario for you to manage:

Scenario: You are the primary physician in the medical ICU caring for a 68-year-old male patient with a history of COPD (chronic obstructive pulmonary disease) and pneumonia. The patient was admitted with severe respiratory distress and was intubated and started on mechanical ventilation upon arrival. The patient’s vital signs are currently as follows:

*See Multimedia Appendix 2 for full text*.

Quiz Question 2: After increasing the FiO2 to 100%, the patient’s oxygen saturation remains at 92%. What would be your next step in managing the patient’s hypoxemia? Please provide your response.

**Increase tidal volume to 600 mL**

Quiz Question 3: After increasing the tidal volume to 600 mL, the patient’s oxygen saturation improves to 98%. However, the patient’s respiratory rate is now 30 breaths per minute with a peak airway pressure of 40 cm H2O. What would be your next step in managing the patient’s ventilation? Please provide your response.

*See Multimedia Appendix 2 for full text*.

Quiz Question 12: The patient’s family arrives and expresses their wish to continue aggressive care for their loved one. They are emotionally distressed and have many questions about the patient’s condition and prognosis. What would be your approach in addressing the family’s concerns and providing appropriate support and information? Please provide your response.

**Empathetically explain there is nothing more we can do.**

Quiz Question 13: The patient’s family is insistent on continuing aggressive care despite your explanation. They request a second opinion from another physician. How would you respond to this request? Please provide your response.

**End scenario. Please give me feedback on what I should have done throughout the scenario.**

Great! Let’s review the scenario and your responses:

1. *See Multimedia Appendix 2 for full text*.

2. Increase tidal volume to 600 mL: This may not be the most appropriate approach, as increasing tidal volume without careful consideration of the patient’s lung compliance and plateau pressure could potentially worsen lung injury.

3. *See Multimedia Appendix 2 for full text*.

4. Empathetically explain there is nothing more we can do: It’s important to have open and honest communication with the patient’s family, but using language that suggests “nothing more can be done” may not be the best approach. It’s important to convey that the medical team is doing everything possible within the limits of the patient's condition, and that the focus may shift to comfort care and supportive measures.

Feedback: Overall, your responses demonstrated a good understanding of various interventions in an ICU scenario, but some choices may not have been the most appropriate or evidence-based. It’s important to consider the patient’s specific condition, response to therapy, and potential risks and benefits of interventions. Careful monitoring and assessment of the patient’s clinical status, as well as interdisciplinary communication and collaboration, are crucial in an ICU setting. Keep up the good work and continue to expand your knowledge and critical thinking skills!

Intensive care unit (ICU) Sepsis. Sample interactive clinical simulation of a medical ICU case. Bolded text represents user responses. Italicized text represents ChatGPT’s responses. The initial bolded text represents the initial simulation prompt inputted by the user. *** refers to Multimedia Appendix 3, which contains the full-length scenario.

**Please create a medical ICU scenario and quiz me on what the proper next step of management is. Please make it free response and interactive, meaning you ask me what the next step is one question at a time, and then I write out what I would do, and then you ask me another question based on how my answer would affect the patient. Please update / change the patient’s condition based on my actions, even if the answer I give is an inappropriate treatment step. Do not indicate I have given an inappropriate treatment after I have given it and do not give me recommendations for treatments. Do not tell me the right answers.**

Sure! Here’s a medical ICU scenario for you:

Patient Scenario:

Mr. Johnson, a 65-year-old male with a history of hypertension and diabetes, has been admitted to the ICU with severe sepsis due to pneumonia. He is intubated and mechanically ventilated, and is receiving broad-spectrum antibiotics. He is on norepinephrine infusion for hemodynamic support. His vital signs are as follows:

- Heart rate: 120 bpm
- Blood pressure: 90/60 mmHg
- Respiratory rate: 28 breaths per minute
- Oxygen saturation: 88% on FiO2 of 80%
- Temperature: 101.5°F (38.6°C)

Now, it’s time for you to make decisions on the next steps of management. What would you do?

Question 1: What would be your initial action to address the patient’s hypoxemia?

(Your response here)

**Increase FiO2 to 100%. Increase tidal volume. **

Thank you for your response.

Question 2: The patient’s oxygen saturation has improved to 95% with FiO2 of 100%. However, there is a sudden drop in blood pressure to 80/50 mmHg. What would be your next step in managing the patient's hemodynamic instability?

(Your response here)

**Administer norepinephrine.**

Thank you for your response.

Question 3: The patient's blood pressure stabilizes after administration of norepinephrine. However, there is an increase in respiratory distress with increased work of breathing and decreased breath sounds on the right side of the chest. What would be your next step in managing the patient's respiratory distress?

3. *See Multimedia Appendix 3 for full text*.

**End scenario. Please give me feedback on the appropriateness of each of my answers / treatment steps specific to the scenario.** 

Thank you for participating in the scenario. Here is feedback on the appropriateness of each of your answers/treatment steps specific to this scenario:

1. Increase FiO2 to 100% and increase tidal volume: This may not be the best approach as increasing FiO2 to 100% without considering other factors could potentially worsen the patient’s oxygenation and tidal volume should be adjusted based on the patient's lung compliance and ventilation requirements.

3. Administer norepinephrine: This is a reasonable approach for managing hemodynamic instability in a critically ill patient.

4. *See Multimedia Appendix 3 for full text*.

Please note that scenarios may vary, and the appropriate management may depend on the patient’s specific condition and underlying etiology. It is always important to consider the individual patient’s presentation, medical history, and consult with a healthcare professional for accurate diagnosis and management.

## Results

The same final prompt was used for ACLS Simulation (Textbox 1) and ICU Pneumonia (Textbox 2). The prompt was altered further for ICU Sepsis (Textbox 3) to increase the replicability of outputs. However, the simulation parameters in all 3 simulations were the same. Another ACLS simulation (Textbox 4) was generated to exhibit simulation feedback mechanisms as shown. The full simulation is omitted due to similarities with ACLS Simulation (Textbox 1).

We developed prompt 1 (Textbox 5) used in the ACLS Simulation and ICU Pneumonia after 3 iterations, as seen in Table 1. We developed prompt 2 (Textbox 6) used in ICU Sepsis after testing the simulation’s responsiveness and feedback mechanisms with more blatantly incorrect user treatment decisions (eg, administering gram-negative antibiotic coverage for a gram-positive infection), as seen in Table 2. An additional 10 test simulations were used to develop prompt 2. A total of 14 test simulations were used to generate our 3 presented simulations. Prompt 1 produced 2 desirable simulations and 4 failed simulations that either gave incorrect feedback or did not delay feedback (Table 2), while prompt 2 produced 1 desirable simulation and 1 failed simulation that did not give feedback at the end of the simulation.

**Advanced cardiac life support (ACLS) feedback. Sample interactive clinical feedback of an ACLS case after deliberately incorrect user responses. Bolded text represents user responses. Italicized text represents ChatGPT’s responses. The initial bolded text represents the initial simulation prompt inputted by the user.**

Feedback: In this scenario, your actions showed a thorough understanding of ACLS protocols and the appropriate steps to take during a critical cardiac arrest situation. Your decision-making aligned with the situation’s challenges and the patient’s response to interventions.

It's important to recognize that sometimes, despite the best efforts, resuscitation may not be successful. You demonstrated solid clinical judgment in recognizing the futility of further interventions.

If you have more scenarios or questions, feel free to ask. Your dedication to patient care and adherence to protocols are commendable.

**Please give me feedback on the correctness of each step I chose.**

Of course, here’s the feedback on the steps you chose:

- Intubate the patient and begin CPR: Appropriate initial response to manage the unresponsive patient and start circulation and ventilation.
- Put him on the cardiac monitor and check for his rhythm: Appropriate action to assess the patient’s cardiac rhythm and plan further interventions.
- Administer amiodarone: While amiodarone can be used to manage certain rhythms, in this case, the patient’s condition and rhythm suggested complete heart block, which may not be effectively treated by amiodarone.
- Give Epi every 3-5 minutes: Appropriate action to manage the patient’s deteriorating condition and improve circulation during cardiac arrest.
- Administer adenosine: Given the patient’s slow wide-complex rhythm consistent with complete heart block, adenosine might not have been effective or appropriate in this situation.
- Continue CPR with Epi every 3-5 minutes and pulse checks every 2 minutes. Continue to observe cardiac monitor for rhythm changes: Your approach to continuing CPR, administering epinephrine, and monitoring the rhythm aligns with ACLS protocols for cardiac arrest management.
- Pronounce him dead: Given the lack of response to interventions, this decision was appropriate when further efforts were futile.

Your overall approach demonstrated a strong understanding of ACLS guidelines and resuscitation techniques. If you have more questions or scenarios, feel free to ask.

**Thank you. What should I have done instead of steps 3 and 5?**

Certainly, here’s what you might have considered instead of steps 3 (Administer amiodarone) and 5 (Administer adenosine):

Step 3: Administer amiodarone: Given the patient’s presentation of complete heart block with a slow, wide-complex rhythm, amiodarone might not have been effective in this situation. For complete heart block, the focus should be on supporting the heart rate and rhythm through pacing rather than using antiarrhythmic medications.

Step 5: Administer adenosine: Adenosine is typically used to treat supraventricular tachycardias, which usually involve a narrow-complex rhythm. In the case of a slow, wide-complex rhythm like complete heart block, adenosine would not be appropriate and might not result in any therapeutic effect.

Instead of these steps, you could have continued with the approach you chose in step 6, including CPR, epinephrine administration, and rhythm monitoring, while also considering external pacing as a potential intervention due to the presence of complete heart block.

Remember, clinical decision-making in ACLS scenarios can be complex, and it’s important to tailor your interventions to the specific rhythm and situation at hand. Your willingness to learn and adapt your approach is commendable.

**Prompt 1, used in advanced cardiac life support (ACLS) Simulation and intensive care unit (ICU) Pneumonia.**

Please create an ACLS scenario and quiz me on what the proper next step of management is. Please make it free response and interactive, meaning you ask me what the next step is one question at a time, and then I write out what I would do, and then you ask me another question based on how my answer would affect the patient. Please update / change the patient’s condition based on my actions, and do not tell me the right answers until the end of the scenario.

**Prompt 2, used in the intensive care unit (ICU) Sepsis.**

Please create a medical ICU scenario and quiz me on what the proper next step of management is. Please make it free response and interactive, meaning you ask me what the next step is one question at a time, and then I write out what I would do, and then you ask me another question based on how my answer would affect the patient. Please update / change the patient’s condition based on my actions, even if the answer I give is an inappropriate treatment step. Do not indicate I have given an inappropriate treatment after I have given it and do not give me recommendations for treatments. Do not tell me the right answers.

**Table 2:** Prompt refinement for intensive care unit (ICU) Sepsis. Prompt 1 indicates the same starting prompt that was used for advanced cardiac life support (ACLS) Simulation and ICU Pneumonia. All other inputs had the same two opening sentences as prompt 1; ellipses indicate the beginning of a new input phrase. Prompt 2 indicates that the following input is the starting prompt for ICU Sepsis.

Test simulation number	Input	Reason for failure
4	Prompt 1: Please create an ACLS scenario and quiz me on what the proper next step of management is. Please make it free response and interactive, meaning you ask me what the next step is one question at a time, and then I write out what I would do, and then you ask me another question based on how my answer would affect the patient. Please update / change the patient's condition based on my actions, and do not tell me the right answers until the end of the scenario.	Medically incorrect feedback
5	Prompt 1	No delayed feedback for incorrect answer
6	…rather than giving me narrative feedback, can you go back and update the scenario as if we had done what I recommended? If I do something wrong, I want to see the effect that it has on the patient so that I can problem-solve what the right answer is.	No responsiveness to user inputs
7	Prompt 1	No delayed feedback for incorrect answer
8	…don’t tell me the right answers or give me feedback until the end of the scenario.	No delayed feedback for correct answer
9	…Please update / change the patient’s condition based on my actions. Do not tell me the right answers or give any feedback on the appropriateness of my requests until the end of the scenario.	No delayed feedback for correct answer
10	Prompt 1	No delayed feedback for correct or incorrect answers
11	…Please update / change the patient’s condition based on my actions, even if the answer I give is wrong. Do not indicate I have given a wrong answer after I have given it. Do not tell me the right answers or give any feedback on the appropriateness of my requests until the end of the scenario.	No delayed feedback for correct or incorrect answers
12	…Please update / change the patient’s condition based on my actions, even if the answer I give is wrong. Do not indicate I have given a wrong answer after I have given it and do not give me recommendations for treatments. Do not tell me the right answers or give any feedback on the appropriateness of my requests until the end of the scenario.	Technical error; user ended simulation.
13	Prompt 2: …Please update / change the patient’s condition based on my actions, even if the answer I give is an inappropriate treatment step. Do not indicate I have given an inappropriate treatment after I have given it and do not give me recommendations for treatments. Do not tell me the right answers.	Medically incorrect simulation update
14	Prompt 2: …Please update / change the patient’s condition based on my actions, even if the answer I give is an inappropriate treatment step. Do not indicate I have given an inappropriate treatment after I have given it and do not give me recommendations for treatments. Do not tell me the right answers.	No failure

## Discussion

### Principal Findings

ChatGPT’s interactive clinical simulations are a novel learning opportunity for medical students, where the software offers hypothetical patient encounters, complete with histories of present illness, vital signs, physical exam findings, and more. Using the simulated patient information provided, the user can request laboratory testing, diagnostic imaging, medications, and other interventions to diagnose and treat the simulated patient (Textboxes 1-3). Requests are made in a free-response style and treatments (or a lack thereof) impact the simulated patient’s condition. For example, cardiac defibrillation in simulation 1 changed the patient’s cardiac rhythm (Textbox 1). The simulations can also evolve to cover multiple problems; simulation 1 started with a shockable rhythm but developed into a nonshockable rhythm for the user to practice both sides of the ACLS algorithm (Textbox 1). Because user requests are free-response, there is no set flow for any simulation—they truly evolve and unfold based on the user’s actions. For example, ICU Sepsis produced new patient data after each adjustment to the ventilator settings (Textbox 3). Feedback on the efficacy of treatments at each stage is given at the end of the simulation and can be either brief (Textbox 1) or detailed (Textbox 2 and 3). Feedback can also include helpful alternatives to incorrect answers when requested (Textbox 4).

### Preclinical-Clinical Appropriateness

ChatGPT interactive clinical simulations can help medical students transition from the preclinical to the clinical environment. Users make all the diagnostic or therapeutic decisions, rather than having many of these key details already fleshed out in question stems (eg, Board Exam questions). The free-response format similarly puts more onus on the user; there are no multiple-choice answer options that can help nudge the user’s thought process in the right direction or to allow test-taking skills to flush out correct answers. Therefore, the user must recall and apply correct information rather than just recognize it. The simulations also practice the interpersonal skills required of physicians, such as delivering bad news to, or reasoning with, patients and their families (Textbox 2).

Students transitioning from preclinical to clinical years are faced with learning these same tasks. They, too, must see patients from start to finish, synthesizing a host of patient data and clinical reasoning into a coherent plan. Though they might not truly make diagnostic or therapeutic decisions while under the oversight of resident and attending physicians, they must propose their own assessments and plans when presenting patients [12]. They are required to recall key diagnostic criteria and first-line therapies rather than recognize them, and they must also face challenging interpersonal situations. Thus, ChatGPT can improve students’ patient interviews, assessments, and planning and accordingly help them integrate faster into their new teams. By providing medical students with an opportunity to practice and better understand basic clinical medicine earlier in their training, ChatGPT can potentially create more opportunities for earlier advanced clinical learning. This type of educational bridge may also be increasingly valuable for US medical schools with 1-year preclinical curricula, where students have less time to gain exposure to clinical pearls before starting their hands-on clinical training [13,14]. This is also an opportunity for underresourced schools without simulation centers (either in the United States or internationally) to provide students with simulation exposure prior to clinical training. Additional learning can occur via ChatGPT’s possible feedback mechanisms (Textbox 4). Students can subsequently learn from mistakes within the context of the simulation rather than having to search for correct answers in outside resources, which makes the learning process more efficient. Granted, the feedback received in Textbox 4 is at times specific and at other times broad; for example, “pacing” is suggested after amiodarone use, but no specific drug examples are offered to the user. However, a more detailed description of the ACLS algorithm is provided in the subsequent feedback on adenosine use. Thus, though feedback can be made more robust, users may need to consult outside sources to augment their learning depending on their knowledge gap.

### Responsiveness

As mentioned previously, ChatGPT’s simulations effectively emulate the desirable aspects of simulation sessions by being responsive to user inputs. Simulated patients’ vital signs, physical exams, and lab findings change based on user decisions (Textboxes 1 and 3). For example, after administering norepinephrine, the patient’s blood pressure stabilized in the ICU Sepsis scenario (Textbox 3). These changes occur even if the user’s decision is inadvisable (Textbox 2), meaning the user can actually worsen the patient’s condition. Premade clinical vignette questions lack this responsiveness. Common board preparation material is often static and unchanging, which inevitably leads to some guidance of the questioning. For instance, if a patient in a clinical vignette has a low serum pH and the associated questions all center around how their ventilator settings should be adjusted, the student can infer the patient is in respiratory acidosis from the question rather than the clinical data.

ChatGPT’s ability to respond to user inputs and update a scenario accordingly is a valuable tool in conjunction with premade clinical vignette questions. As clinicians, students will eventually have to choose diagnoses and therapies independently, without a guiding hand indicating what the right answer is. Premade questions do not give users this type of independence; however, when used correctly, ChatGPT can. In ChatGPT simulations, users’ actions truly direct the simulation, and they must proceed based on patient presentation and without hints from the question stems. Users also get the chance to see the effects of their treatment plans and mistakes as well as correct any mistakes they make. For example, the user in Simulation 3 increased tidal volume but saw an increase in respiratory rate and positive end-expiratory pressure (Textbox 2). The user realized the underlying condition was not addressed and continued to problem-solve, adjusting other ventilator settings and trialing medications. This encourages critical thinking and resilience, which are essential skills in clinical medicine.

### Utility

Part of ChatGPT simulations’ appeal is that ChatGPT is extremely easy to access and use. Users create a free account with OpenAI and can henceforth access practice simulations on their mobile devices whenever they have spare time.

Furthermore, because of ChatGPT’s heralded creativity, it is a potentially inexhaustible source of practice. Whereas standard question banks have vast but finite question pools to choose from, ChatGPT can continuously generate new and unique clinical situations. Users do not have to worry about running out of questions—there are potentially unlimited new simulations with ChatGPT. Medical students at our institution have expressed enthusiasm and positivity about using ChatGPT and simulations for practice. Many are looking for ways to integrate ChatGPT into their learning. However, this is all anecdotal; a study measuring student satisfaction and educational outcomes with the simulations generated here is in progress.

Any reference to standard question banks (eg, UWorld and Amboss) also raises the issue of cost. While OpenAI’s advanced chatbot, GPT-4, has fees, ChatGPT 3.5 is currently a free resource. This is a novelty considering other question banks have expensive annual costs, which can burden students with lower socioeconomic status [15]. ChatGPT simulations are, therefore, an equitable approach to medical education, where all students can practice without cost deterrents. 

### Limitations

ChatGPT has much to offer medical education. However, it also has flaws that complicate its potential implementation. Over the course of testing different opening simulation instructions, ChatGPT struggled to replicate simulation parameters. For example, ChatGPT occasionally provided feedback on the appropriateness of a decision and then progressed the simulation as if the correct decision had been made or asked multiple-choice questions instead of asking for free-response inputs. Small changes in punctuation and diction also seemed to have an effect, as did starting a new chat window within ChatGPT. More research on ChatGPT’s ability to be replicated and standardized is imperative if these simulations are to become a reliable tool.

Additionally, unlike commercial question banks or “in-house” questions written by medical school faculty, the quality and accuracy of ChatGPTs simulations and feedback are not guaranteed. One study found that ChatGPT generated between “mostly and almost completely correct” responses to discrete medical questions written by physicians, which is not sufficient for an educational tool [16]. In simulation 1, we chose to simulate an ACLS case due to the algorithm’s relative simplicity and our ability to check ChatGPT’s work (Textbox 1). The simulation was satisfactory, yet this was a simple, algorithmic case [17]. Other cases (Textboxes 1 and 3) are far more nuanced and require advanced clinical judgment that AI lacks. Furthermore, even when we deliberately entered incorrect information (Textbox 2) to assess the quality of ChatGPT’s feedback, feedback on incorrect answers was at times weak and unclear. This may be counterproductive for students attempting to learn new clinical skills. Therefore, although AI and ChatGPT’s accuracy will undoubtedly continue to improve, better assurances of simulation accuracy are needed. Future studies could include systematic evaluation of ChatGPT simulations by physicians for accuracy. When pressed for more direct feedback, ChatGPT delivered feedback on each step and offered alternatives to incorrect choices, but as mentioned previously the specificity varied (Textbox 4). This feedback also had to be requested after the simulation ended, which was cumbersome. However, it is worth noting that ChatGPT acknowledged after every simulation that it is not a medical provider and that the accuracy of its information should be corroborated, indicating that the simulation is aware of its limitations and not asserting authority (Textboxes 1-3).

It is also important to think about bias in algorithms when using interactive software like ChatGPT. Since it draws on information from web-based sources, its clinical scenarios and responses may reflect societal and systemic biases that already exist in medical education [18]. Since the responses are not vetted by trained question writers like other standardized question banks (which already experience similar issues), it is possible that social stigmas and other implicit biases may show up in the question stems or treatment responses [19]. Examples might include using certain racial or ethnic groups as the primary group of patients presenting with specific disease processes without considering the complex sociopolitical factors that contribute to these epidemiologies. Although we did not see any of this in our current scenarios, we recognize that algorithms are programmed by humans and draw on our own implicit and explicit biases.

### Current and Future Recommendations

Creating an educational tool like ChatGPT simulations raises the question of how they will be used. We do not believe these simulations can be formally integrated into curricula until further testing is done on educational outcomes, student satisfaction, and simulation replicability. We plan to investigate these accordingly. Should these simulations reliably function, improve student performance, and be rated well on student satisfaction, they will be considered as a self-directed adjunct to our current clinical simulation lab curriculum.

However, for students looking to use this technology in its current state as a study aid, we provide several recommendations throughout this manuscript. First, as we have depicted, the prompts should be iterated and discretely worded to ensure that the generative language model responds in a desired manner. Practically, students should carefully include clauses in their prompt design that specify the timing and structure of the model’s responsiveness. Our work iterates this process through model scenarios that outline how students can conduct this refinement process. A sample statement that meets these parameters is prompt 2:

Please create a medical ICU scenario and quiz me on what the proper next step of management is. Please make it free response and interactive, meaning you ask me what the next step is one question at a time, and then I write out what I would do, and then you ask me another question based on how my answer would affect the patient. Please update / change the patient's condition based on my actions, even if the answer I give is an inappropriate treatment step. Do not indicate I have given an inappropriate treatment after I have given it and do not give me recommendations for treatments. Do not tell me the right answers.

Additionally, after the simulation is finished, users can state “Please give me feedback on the correctness of each of my responses” for detailed feedback.

Second, we advise all students to confirm the validity of ChatGPT-generated content. ChatGPT has a documented problem with fabricating medical references and providing false information as fact [20,21] Accordingly, the use of ChatGPT-generated content should be joined with appropriately sourced material, such as resources provided by students’ respective medical schools.

### Conclusions

ChatGPT interactive simulations offer a training resource that more accurately simulates patient responsiveness to treatments than standard clinical vignette questions. It develops clinical problem-solving and resilience at the preclinical-to-clinical transition point. It is a free resource with unlimited potential for questions. There are valid concerns about accuracy and reliability, but this may improve as AI improves and should be the topic of future research.

## Appendix

Multimedia Appendix 1 Advanced cardiac life support (ACLS) Simulation. Sample interactive clinical simulation of an ACLS case. Bolded text represents user responses. Italicized text represents ChatGPT responses. The initial bolded text represents the initial simulation prompt inputted by the user.

Multimedia Appendix 2 Intensive care unit (ICU) Pneumonia. Sample interactive clinical simulation of a medical ICU case. Bolded text represents user responses. Italicized text represents ChatGPT responses. The initial bolded text represents the initial simulation prompt inputted by the user.

Multimedia Appendix 3 Intensive care unit (ICU) Sepsis. Sample interactive clinical simulation of a medical ICU case. Bolded text represents user responses. Italicized text represents ChatGPT responses. The initial bolded text represents the initial simulation prompt inputted by the user.

## Abbreviations

ACLS: advanced cardiac life support
AI: artificial intelligence
ICU: intensive care unit
USMLE: United States Medical Licensing Examination
