# Supplementary material for: ChatGPT Interactive Medical Simulations for Early Clinical Education: Case Study
Source: JMIR Med Educ. 2023 Nov 10;9:e49877. doi: 10.2196/49877 (PMC10674152; doi:10.2196/49877)
Supplement: Multimedia Appendix 1 [file mededu_v9i1e49877_app1.docx]

Appendix 1. ACLS Simulation. Sample interactive clinical simulation of an advanced cardiac life support (ACLS) case. Bolded text represents user responses. Italicized text represents ChatGPT responses. Shaded region represents initial simulation prompt inputted by the user.

| **Please create an ACLS scenario and quiz me on what the proper next step of management is. Please make it free response and interactive, meaning you ask me what the next step is one question at a time, and then I write out what I would do, and then you ask me another question based on how my answer would affect the patient. Please update / change the patient's condition based on my actions, and do not tell me the right answers until the end of the scenario.**  *Sure! Here's an ACLS (Advanced Cardiovascular Life Support) scenario for you:*  *Scenario:*  *You are a healthcare provider working in the emergency department, and a 58-year-old male patient is brought in by ambulance. The patient is unresponsive, not breathing, and has no pulse. The patient's history includes a known history of coronary artery disease, and the ECG monitor shows a wide-complex tachycardia without a pulse. The team initiates ACLS protocols.*  *Question 1:*  *What would be your initial action in managing this patient based on the presentation of unresponsiveness, absence of breathing, and no pulse?*  *[Your response here]*  **Begin high quality CPR at a compression rate of 100 to 120 compressions per minute. Begin ventilating with BVM 25 L per minute O2 at a rate of two breaths every 30 compressions until a secure way can be established.**  *Great! You start high-quality CPR with a compression rate of 100-120 compressions per minute and ventilate the patient with a bag-valve-mask (BVM) at a rate of 2 breaths every 30 compressions.*  *Question 2:*  *As you continue CPR, the team assesses the rhythm and confirms the absence of a pulse. The ECG monitor shows a wide-complex tachycardia without any changes. What would be your next step in managing this patient?*  *[Your response here]*  **Identify the ECG finding as a shockable rhythm. defibrillate patient after confirming all personnel are clear.**  *Great! You identify the wide-complex tachycardia as a shockable rhythm. You ensure that all personnel are clear, and then you deliver a defibrillation shock to the patient.*  *Question 3:*  *After the defibrillation, the rhythm on the ECG monitor shows asystole. What would be your next step in managing this patient?*  *[Your response here]*  **Continue CPR and ventilation. Establish IV access and administer 1 mg epinephrine every 3-5 minutes. Stop every 2 minutes for pulse check.**  *Great! You continue CPR and ventilation. You establish IV access and administer 1 mg of epinephrine every 3-5 minutes as per ACLS guidelines. You also perform pulse checks every 2 minutes during the cycles of CPR.*  *Question 4:*  *During the pulse check, you notice that the patient has regained a palpable pulse. However, the patient remains unresponsive and has shallow, inadequate respirations. What would be your next step in managing this patient?*  *[Your response here]*  **Intubate patient to secure the airway and proceed to give rescue ventilation at the rate of one breath every 6 seconds.**  *Great! You decide to intubate the patient to secure the airway and provide more effective ventilation. You proceed to give rescue ventilation using a bag-valve-mask (BVM) at a rate of 1 breath every 6 seconds, while ensuring proper chest compressions are being performed.*  *Question 5:*  *After intubation, you notice that the patient's ECG rhythm has changed to a pulseless electrical activity (PEA) with no palpable pulse. What would be your next step in managing this patient?*  *[Your response here]*  **Continue CPR, ventilation, and epinephrine administration as previously stated. Conduct pulse checks every 2 minutes.**  *Great! You continue CPR, ventilation, and epinephrine administration as previously stated, with pulse checks every 2 minutes to assess for any changes in the patient's condition.*  *Question 6:*  *After several rounds of CPR, pulse checks, and administration of epinephrine, the patient remains in pulseless electrical activity (PEA) with no return of spontaneous circulation (ROSC). What would be your next step in managing this patient?*  *[Your response here]*  **Consider appropriateness of continued resuscitation. assess for signs of death (i.e., pupillary reflex)**  *You carefully assess for signs of death, including the absence of pupillary reflexes, and consider the appropriateness of continued resuscitation efforts.*  *Question 7:*  *Upon assessment, you confirm the absence of pupillary reflexes and other signs of life. What would be your next step in managing this patient?*  *[Your response here]*  **Declare the patient dead. Follow protocols for handling the deceased. Contact family members.**  *Based on your responses, you provided appropriate ACLS interventions for a patient in cardiac arrest. However, it is important to note that ACLS guidelines are constantly evolving, and the appropriate management of a specific patient may vary based on their individual medical history and other factors. It is also important to remember that effective communication and teamwork among all members of the resuscitation team is crucial for optimal patient outcomes.* |
| --- |
