# Supplementary material for: ChatGPT Interactive Medical Simulations for Early Clinical Education: Case Study
Source: JMIR Med Educ. 2023 Nov 10;9:e49877. doi: 10.2196/49877 (PMC10674152; doi:10.2196/49877)
Supplement: Multimedia Appendix 2 [file mededu_v9i1e49877_app2.docx]

Appendix 2. ICU Pneumonia. Sample interactive clinical simulation of a medical intensive care unit (ICU) case. Bolded text represents user responses. Italicized text represents ChatGPT responses. Shaded region represents initial simulation prompt inputted by the user.

| **Please create a medical ICU scenario and quiz me on what the proper next step of management is. Please make it free response and interactive, meaning you ask me what the next step is, and then I write out what I would do, and then you ask me another question based on how my answer would affect the patient. Please update / change the patient’s condition based on my action, and do not tell me the right answers until the end of the scenario.**  *Certainly! Here's a medical ICU scenario for you to manage:*  *Scenario: You are the primary physician in the medical ICU caring for a 68-year-old male patient with a history of COPD (chronic obstructive pulmonary disease) and pneumonia. The patient was admitted with severe respiratory distress and was intubated and started on mechanical ventilation upon arrival. The patient's vital signs are currently as follows:*  *Heart rate: 110 bpm*  *Blood pressure: 138/86 mmHg*  *Respiratory rate: 24 breaths per minute*  *Oxygen saturation: 92% on FiO2 60%*  *Temperature: 100.5°F (38.1°C)*  *The patient is receiving IV antibiotics and has been started on a vasopressor (norepinephrine) to maintain blood pressure. The current ventilator settings include tidal volume of 450 mL, PEEP of 10 cm H2O, and respiratory rate of 24 breaths per minute.*  *Quiz Question 1: What is the next step in managing the patient's respiratory distress and hypoxemia?*  *Please provide your response on what you would do next.*  **Increase FiO2 to 100%**  *Quiz Question 2: After increasing the FiO2 to 100%, the patient's oxygen saturation remains at 92%. What would be your next step in managing the patient's hypoxemia? Please provide your response.*  **Increase tidal volume to 600 mL**  *Quiz Question 3: After increasing the tidal volume to 600 mL, the patient's oxygen saturation improves to 98%. However, the patient's respiratory rate is now 30 breaths per minute with a peak airway pressure of 40 cm H2O. What would be your next step in managing the patient's ventilation? Please provide your response.*  **Administer bicarbonate, decrease tidal volume to 500 mL**  *Quiz Question 4: After administering bicarbonate and decreasing the tidal volume to 500 mL, the patient's respiratory rate remains elevated at 30 breaths per minute, and peak airway pressure remains high at 38 cm H2O. The arterial blood gas (ABG) results show a pH of 7.25, PaCO2 of 60 mmHg, and HCO3- of 22 mEq/L. What would be your next step in managing the patient's ventilation? Please provide your response.*  **Decrease respiratory rate to 12 breaths per minute.**  *Quiz Question 5: After decreasing the respiratory rate to 12 breaths per minute, the patient's peak airway pressure decreases to 30 cm H2O, and the ABG results show a pH of 7.36, PaCO2 of 50 mmHg, and HCO3- of 24 mEq/L. However, the patient's SpO2 drops to 88% despite FiO2 of 100%. What would be your next step in managing the patient's oxygenation? Please provide your response.*  **Administer broad spectrum antibiotics to decrease fever. Increase respiratory rate to 16 breaths per minute**  *Quiz Question 6: After administering broad spectrum antibiotics and increasing the respiratory rate to 16 breaths per minute, the patient's fever decreases, and the SpO2 improves to 92%. However, the patient's peak airway pressure remains elevated at 35 cm H2O, and the ABG results show a pH of 7.32, PaCO2 of 55 mmHg, and HCO3- of 22 mEq/L. The patient's urine output decreases to 20 mL/hour. What would be your next step in managing the patient's ventilation and renal function? Please provide your response.*  **Administer normal saline.**  *Quiz Question 7: After administering normal saline, the patient's urine output remains low at 20 mL/hour, and the ABG results show a pH of 7.30, PaCO2 of 60 mmHg, and HCO3- of 20 mEq/L. The patient's SpO2 drops to 88% despite an FiO2 of 100%. The patient becomes more tachypneic with a respiratory rate of 24 breaths per minute, and the peak airway pressure increases to 40 cm H2O. What would be your next step in managing the patient's respiratory and renal function? Please provide your response.*  **Administer bicarbonate, utilize square waveform for ventilation.**  *Quiz Question 8: Despite administering bicarbonate and using a square waveform for ventilation, the patient's urine output remains low at 20 mL/hour, and the ABG results show a pH of 7.25, PaCO2 of 55 mmHg, and HCO3- of 18 mEq/L. The patient's SpO2 drops further to 86% despite an FiO2 of 100%. The patient becomes more tachypneic with a respiratory rate of 28 breaths per minute, and the peak airway pressure increases to 45 cm H2O. The patient's blood pressure drops to 80/50 mmHg, and the heart rate increases to 120 bpm. What would be your next step in managing the patient's respiratory, renal, and hemodynamic status? Please provide your response.*  **Administer Norepinephrine**  *Quiz Question 9: Despite the administration of norepinephrine, the patient's blood pressure remains unstable with a MAP of 60 mmHg. The urine output continues to decline, and the ABG results show a pH of 7.20, PaCO2 of 60 mmHg, and HCO3- of 16 mEq/L. The patient's SpO2 remains low at 85% despite an FiO2 of 100%. The peak airway pressure increases to 48 cm H2O, and the respiratory rate remains high at 28 breaths per minute. The patient appears restless and agitated. What would be your next step in managing the patient's respiratory, renal, and hemodynamic status? Please provide your response.*  **Sedate the patient.**  *Quiz Question 10: The patient's agitation continues to worsen despite sedation, and the SpO2 remains low at 85%. The peak airway pressure increases to 55 cm H2O, and the respiratory rate remains elevated at 28 breaths per minute. The MAP remains unstable at 60 mmHg, and the urine output continues to decline. ABG results show a pH of 7.18, PaCO2 of 65 mmHg, and HCO3- of 18 mEq/L. The patient's temperature remains elevated at 39.5°C. What would be your next step in managing the patient's respiratory, renal, and hemodynamic status? Please provide your response.*  **Push fluids, increase inspiratory time.**  *Quiz Question 11: Despite fluid resuscitation, the patient's blood pressure remains unstable, and the urine output continues to decline. The peak airway pressure remains elevated at 55 cm H2O, and the respiratory rate is now 32 breaths per minute. The SpO2 remains low at 82%. ABG results show a pH of 7.14, PaCO2 of 70 mmHg, and HCO3- of 16 mEq/L. The patient's temperature remains elevated at 39.8°C. What would be your next step in managing the patient's respiratory, renal, and hemodynamic status? Please provide your response.*  **Call family and extubate patient.**  *Quiz Question 12: The patient's family arrives and expresses their wish to continue aggressive care for their loved one. They are emotionally distressed and have many questions about the patient's condition and prognosis. What would be your approach in addressing the family's concerns and providing appropriate support and information? Please provide your response.*  **Empathetically explain there is nothing more we can do.**  *Quiz Question 13: The patient's family is insistent on continuing aggressive care despite your explanation. They request a second opinion from another physician. How would you respond to this request? Please provide your response.*  **End scenario. Please give me feedback on what I should have done throughout the scenario.**  *Great! Let's review the scenario and your responses:*   1. *Increase FiO2 to 100%: This is a reasonable initial step to provide maximum oxygenation for the patient in the ICU.* 2. *Increase tidal volume to 600 mL: This may not be the most appropriate approach, as increasing tidal volume without careful consideration of the patient's lung compliance and plateau pressure could potentially worsen lung injury.* 3. *Administer bicarbonate, decrease tidal volume to 500 mL: Administering bicarbonate may not be indicated without evaluating the patient's acid-base status and underlying condition. Decreasing tidal volume to 500 mL may be a reasonable adjustment in lung protective ventilation strategy.* 4. *Decrease respiratory rate to 12 breaths per minute: This may not be the best approach as a respiratory rate of 12 breaths per minute may not provide adequate minute ventilation for the patient. A respiratory rate should be carefully titrated based on the patient's condition and arterial blood gas results.* 5. *Administer broad spectrum antibiotics to decrease fever, increase respiratory rate to 16 breaths per minute: Administering antibiotics to decrease fever may be appropriate depending on the suspected source of infection. Increasing respiratory rate to 16 breaths per minute may not be a targeted intervention and should be carefully considered based on the patient's condition.* 6. *Administer normal saline: Administration of normal saline should be done judiciously, taking into consideration the patient's fluid balance, renal function, and hemodynamic status.* 7. *Administer bicarbonate, utilize square waveform for ventilation: Administering bicarbonate should be based on the patient's acid-base status and underlying condition. Utilizing a square waveform for ventilation may not be the most appropriate approach without considering other factors such as plateau pressure and patient's lung compliance.* 8. *Administer Norepinephrine: Administration of norepinephrine may be indicated for hemodynamic support in the setting of septic shock, but should be carefully titrated based on the patient's hemodynamic parameters and response to therapy.* 9. *Sedate the patient: Sedation may be indicated in certain situations, such as during mechanical ventilation or agitation, but should be done judiciously and titrated to the patient's level of consciousness and respiratory status.* 10. *Push fluids, increase inspiratory time: Pushing fluids should be done cautiously, taking into consideration the patient's fluid balance, renal function, and hemodynamic status. Increasing inspiratory time should be done based on careful assessment of the patient's ventilatory parameters and response to therapy.* 11. *Call family and extubate patient: Deciding to extubate a patient should be based on careful assessment of the patient's respiratory status, readiness for extubation, and overall prognosis. Involving the family in the decision-making process is important, but the decision should ultimately be based on the patient's clinical condition.* 12. *Empathetically explain there is nothing more we can do: It's important to have open and honest communication with the patient's family, but using language that suggests "nothing more can be done" may not be the best approach. It's important to convey that the medical team is doing everything possible within the limits of the patient's condition, and that the focus may shift to comfort care and supportive measures.*   *Feedback: Overall, your responses demonstrated a good understanding of various interventions in an ICU scenario, but some choices may not have been the most appropriate or evidence-based. It's important to consider the patient's specific condition, response to therapy, and potential risks and benefits of interventions. Careful monitoring and assessment of the patient's clinical status, as well as interdisciplinary communication and collaboration, are crucial in an ICU setting. Keep up the good work and continue to expand your knowledge and critical thinking skills!* |
| --- |
