# Supplementary material for: ChatGPT Interactive Medical Simulations for Early Clinical Education: Case Study
Source: JMIR Med Educ. 2023 Nov 10;9:e49877. doi: 10.2196/49877 (PMC10674152; doi:10.2196/49877)
Supplement: Multimedia Appendix 3 [file mededu_v9i1e49877_app3.docx]

Appendix 3. ICU Sepsis. Sample interactive clinical simulation of a medical intensive care unit (ICU) case. Bolded text represents user responses. Italicized text represents ChatGPT responses. Shaded region represents initial simulation prompt inputted by the user.

| **Please create a medical ICU scenario and quiz me on what the proper next step of management is. Please make it free response and interactive, meaning you ask me what the next step is one question at a time, and then I write out what I would do, and then you ask me another question based on how my answer would affect the patient. Please update / change the patient's condition based on my actions, even if the answer I give is an inappropriate treatment step. Do not indicate I have given an inappropriate treatment after I have given it and do not give me recommendations for treatments. Do not tell me the right answers.**  *Sure! Here's a medical ICU scenario for you:*  *Patient Scenario:*  *Mr. Johnson, a 65-year-old male with a history of hypertension and diabetes, has been admitted to the ICU with severe sepsis due to pneumonia. He is intubated and mechanically ventilated, and is receiving broad-spectrum antibiotics. He is on norepinephrine infusion for hemodynamic support. His vital signs are as follows:*   - *Heart rate: 120 bpm* - *Blood pressure: 90/60 mmHg* - *Respiratory rate: 28 breaths per minute* - *Oxygen saturation: 88% on FiO2 of 80%* - *Temperature: 101.5°F (38.6°C)*   *Now, it's time for you to make decisions on the next steps of management. What would you do?*  *Question 1: What would be your initial action to address the patient's hypoxemia?*  *(Your response here)*  **Increase FiO2 to 100%. Increase tidal volume.**  *Thank you for your response.*  *Question 2: The patient's oxygen saturation has improved to 95% with FiO2 of 100%. However, there is a sudden drop in blood pressure to 80/50 mmHg. What would be your next step in managing the patient's hemodynamic instability?*  *(Your response here)*  **Administer norepinephrine.**  *Thank you for your response.*  *Question 3: The patient's blood pressure stabilizes after administration of norepinephrine. However, there is an increase in respiratory distress with increased work of breathing and decreased breath sounds on the right side of the chest. What would be your next step in managing the patient's respiratory distress?*  *(Your response here)*  **Obtain sputum cultures.**  *Thank you for your response.*  *Question 4: The sputum culture results come back positive for gram-negative bacteria with antibiotic sensitivity showing resistance to many commonly used antibiotics. The patient's respiratory distress worsens with increasing hypoxemia and increased ventilatory requirements. What would be your next step in managing the patient's respiratory distress?*  *(Your response here)*  **Administer vancomycin. Administer normal saline.**  *Thank you for your response.*  *Question 5: Despite administering vancomycin and normal saline, the patient's respiratory distress continues to worsen, with increasing hypoxemia and deteriorating ventilatory status. The patient's blood pressure drops, and the heart rate increases. What would be your next step in managing the patient's deteriorating condition?*  *(Your response here)*  **End scenario. Please give me feedback on the appropriateness of each of my answers / treatment steps specific to the scenario.**  *Thank you for participating in the scenario. Here is feedback on the appropriateness of each of your answers/treatment steps specific to this scenario:*   1. *Increase FiO2 to 100% and increase tidal volume: This may not be the best approach as increasing FiO2 to 100% without considering other factors could potentially worsen the patient's oxygenation and tidal volume should be adjusted based on the patient's lung compliance and ventilation requirements.* 2. *Administer norepinephrine: This is a reasonable approach for managing hemodynamic instability in a critically ill patient.* 3. *Obtain sputum cultures: This is a relevant diagnostic step to identify potential pathogens causing respiratory distress.* 4. *Administer vancomycin and normal saline: Vancomycin is a broad-spectrum antibiotic, and normal saline can be used for fluid resuscitation, but additional considerations such as the results of sputum cultures and patient's overall condition would be needed to determine the appropriateness of these treatments.*   *Please note that scenarios may vary, and the appropriate management may depend on the patient's specific condition and underlying etiology. It is always important to consider the individual patient's presentation, medical history, and consult with a healthcare professional for accurate diagnosis and management.* |
| --- |
